# Guidance on validation and qualification of processes and operations involving radiopharmaceuticals

**DOI:** 10.1186/s41181-017-0025-9

**Published:** 2017-06-29

**Authors:** S. Todde, P. Kolenc Peitl, P. Elsinga, J. Koziorowski, V. Ferrari, E. M. Ocak, O. Hjelstuen, M. Patt, T. L. Mindt, M. Behe

**Affiliations:** 1 University of Milano-Bicocca, Tecnomed Foundation, 20900 Monza, Italy; 2 Department of Nuclear Medicine, University Medical Centre Ljubljana, 1000 Ljubljana, Slovenia; 3 University Medical Center Groningen, University of Groningen, 9700 RB Groningen, The Netherlands; 4 Department of Radiology and Department of Medical and Health Sciences, Linköping University, Linköping, Sweden; 5 GE Healthcare, Amersham, UK; 6 Faculty of Pharmacy, Department of Pharmaceutical Technology, Istanbul University, 34116 Beyazit, Istanbul Turkey; 7 Institute for Energy Technology, Instituttveien 18, PO Box 40, 2027 Kjeller, Norway; 8 Department for Nuclear Medicine, Radiochemistry, Liebigstrasse 18, 04103 Leipzig, Germany; 9 Ludwig Boltzmann Institute Applied Diagnostics, General Hospital Vienna, Nuklearmedizin, Vienna, Austria; 10 Department of Biomedical Imaging and Image Guided Therapy, Division of Nuclear Medicine, Medical University of Vienna, Vienna, Austria; 11 Center for Radiopharmaceutical Sciences ETH-PSI-USZ Paul-Scherrer-Institute, 5232 Villigen-PSI, Switzerland

**Keywords:** Validation, Qualification, Risk Assessment, Radiopharmaceuticals

## Abstract

**Background:**

Validation and qualification activities are nowadays an integral part of the day by day routine work in a radiopharmacy. This document is meant as an Appendix of Part B of the EANM “Guidelines on Good Radiopharmacy Practice (GRPP)” issued by the Radiopharmacy Committee of the EANM, covering the qualification and validation aspects related to the small-scale “in house” preparation of radiopharmaceuticals. The aim is to provide more detailed and practice-oriented guidance to those who are involved in the small-scale preparation of radiopharmaceuticals which are not intended for commercial purposes or distribution.

**Results:**

The present guideline covers the validation and qualification activities following the well-known “validation chain”, that begins with editing the general Validation Master Plan document, includes all the required documentation (e.g. User Requirement Specification, Qualification protocols, etc.), and leads to the qualification of the equipment used in the preparation and quality control of radiopharmaceuticals, until the final step of Process Validation.

**Conclusions:**

A specific guidance to the qualification and validation activities specifically addressed to small-scale hospital/academia radiopharmacies is here provided. Additional information, including practical examples, are also available.

**Electronic supplementary material:**

The online version of this article (doi:10.1186/s41181-017-0025-9) contains supplementary material, which is available to authorized users.

## Definitions

### Good radiopharmacy practice

Good radiopharmacy practice is described in the “Guidelines on Current Good Radiopharmacy Practice (cGRPP)” issued by the Radiopharmacy Committee of the EANM.

### Radiopharmaceutical

A radiopharmaceutical (RP) is any medicinal product which, when ready for use, contains one or more radionuclides (radioactive isotopes) included for a medicinal purpose.

### Small-scale radiopharmaceutical

A small-scale radiopharmaceutical is any in-house radiopharmaceutical not intended for commercial purposes or distribution prepared on a small scale (for PET, SPECT or therapeutic applications), including extemporaneous preparations based on generators and kits, and simple kit preparation based on SPC.

### Small-scale radiopharmacy

A small-scale radiopharmacy is a facility where the small-scale preparation of radiopharmaceuticals is carried out in accordance with national regulations. The term small-scale radiopharmacy is not related to the physical size of the facility, but only to the kind of radiopharmaceutical preparation performed.

### Preparation

Preparation includes all operations involved in the purchase of materials and products, production, QC, release and storage of a medicinal product.

### Finished product

A finished product is a medicinal product which has undergone all stages of production, including QC and product/batch release, packaging in its final container and proper labelling.

### Automated module

An automated module is a device able to perform automatically a sequence of operations needed in the preparation of radiopharmaceuticals. An automated module can be commercial or custom made. It consists of two assembled parts: a mechanical part and a chemistry part.

The mechanical part consists of an assembly of electric and/or pneumatic, linear and/or circular actuators, power supplies, pumps, coolers, heaters, sensors for monitoring different parameters (such as temperature, pressure, flow, radioactivity) or any other physical device not in direct contact with chemicals.

The chemistry part is an interconnected network of containers in which gaseous, liquid and/or solid reagents and components can be moved, mixed and/or transformed to obtain the desired product. The mechanical part and the chemistry part are connected to each other. The chemistry part can be permanent (non-disposable device) or single use (disposable device, or “cassette”).

### SOP

SOP, or Standard Operating Procedure(s) are documents which provide instructions, in a clear and concise form, to perform a specific task. They deal with all the operations and steps involved in the lifecycle of the preparation of a radiopharmaceutical.

### Validation

Validation is the action of proving that any procedure, process, equipment, material, activity or system actually leads to the expected results (World Health Organization).

### Qualification

Action of proving and documenting that any premises, systems and equipment are properly installed, and/or work correctly and lead to the expected results. Qualification is often a part (the initial stage) of validation, but the individual qualification steps alone do not constitute process validation (World Health Organization).

### Validation protocol

A document which contains all the information required to perform the validation of an intended instrument / method / process.

### Design Qualification (DQ)

DQ is aimed to verify that the system / instrument has been designed suitably for the intended purpose. In particular:

- the design meets the user requirement specification (URS);
- the design complies with all the applicable guidelines and standards
- the design complies with the validation master plan (VMP)

### Installation Qualification (IQ)

IQ is aimed to verify that the facility / system / instrument has been installed correctly, based on the manufacturer’s recommendations and/or the approved specifications of the User.

### Operational Qualification (OQ)

OQ is aimed to verify that the facility / system / instrument are operating properly, and that the response of critical components (e.g. sensors) match with the intended values and within the desired range.

### Performance Qualification (PQ)

The goal of PQ is to verify that the facility / system / instrument performs properly and reproducibly in the intended routine conditions set for the specific preparation process, and using approved methods.

### Cleaning validation

Cleaning validation has the purpose to demonstrate that the cleaning of a facility / system / equipment, or those parts of it which come into contact with the finished product or with reagents / solvents during the preparation process, is suitable for the intended purposes, and that residues (chemical, radiochemical, microbiological, cleaning agents) are removed below a defined level by the cleaning procedure.

### Process validation

Process Validation (PV) has to be intended as a mean to establish that all the process parameters that bring to the preparation of the intended RPs and their quality characteristics are consistently and reproducibly met.

### Validation Summary Report (VSR)

VSR is the final document that summarizes the whole protocol results and comments/opinions about their suitability.

### User Requirement Specification (URS)

A set of specifications, that may be related to production/QC equipment, as well as to the whole facility or parts of it such as utilities or systems/sub-systems, defined by the User and that represent a useful reference for the their design and/or purchase, and during the validation activities.

### Validation Master Plan (VMP)

VMP is a general document that summarizes validation policy and all the intended validation / qualification activities, together with a description of the facility and organisational structure.

The present EANM guidance covers the qualification and validation aspects intertwined with the preparation of small-scale radiopharmaceuticals. It concerns the preparation of radiopharmaceuticals which are not intended for commercial purposes or distribution.

## Background

Validation is the action of proving that any procedure, process, equipment, material, activity or system actually leads to the expected results, with the aim to contribute to guarantee the quality of a (radio) pharmaceutical. The concept of qualification is very similar to that of validation, but while the former is more general and relies on a broad range of activities, the latter is more “practical” and indicates the actions and operations aimed to demonstrate that a system / equipment is properly installed, works correctly and leads to the expected results. Qualification may be considered as a part of validation. General Principles on Validation and Qualification are outlined in different important reference documents, the most important and relevant of which, for professionals operating within the European Union, is the Annex 15 (EU) of Good Manufacturing Practice (GMP) guidelines, that apply to the manufacturing of medicinal products aimed to obtain a Marketing Authorization, and in general to those who are requested to comply with GMP. Annex 15 has been recently revised, and most recent version came into operation on 1st October 2015. Other useful guidelines have been released by Institutions such as World Health Organization (WHO) (World Health Organization) or the US Food and Drug Administration (FDA) (FDA Guidance for industry), or even by instrumentation suppliers (Agilent et al. 2017), the latter being usually addressed to specific proprietary technology, while the former are typically conceived as general guidance principles for industry. Although principles described in the above documents are generally applicable to any process, equipment, system or facility, their practical implementation in the preparation and quality controls of radiopharmaceuticals may require adaptations that meet the peculiar nature of the RPs themselves and of the equipment used for their preparation. Another important issue related to the validation concept is the validation of analytical methods, whose general principles are outlined in ICH Q(2) R1 – Note for Guidance on validation of analytical procedures: text and methodology (ICH guideline), which define the type of analytical methods to be validated and set parameters of concern and acceptance criteria to be considered. The same considerations stated above apply: ICH guidelines are very general and capable to embrace a broad range of analytical procedures, including those procedures specifically developed for the quality control of radiopharmaceuticals; however, the intrinsic nature of radioactivity, which decreases with time following the decay law, and the physical characteristics of the detection of radioactivity, prompt for specific validation protocols. Only a brief, general description of the principles of validation of analytical methods will be given in this text; indeed, due to the complexity and variety of the involved procedures, instrumentation, etc., they will be the subject of a separate, dedicated guidance document.

It has to be underlined here that validation may ultimately be considered as a useful way to increase reliability and prevent deviations and out of specification results in the day by day operation in the radiopharmaceutical preparation process, as it is aimed to guarantee that processes / procedures / equipment work correctly and lead to the expected results.

As stated above, the aim of this guideline is to provide more detailed and practice-oriented guidance to those professionals who are involved in the small-scale preparation of radiopharmaceuticals, not intended for commercial purposes or distribution.

## Validation Master Plan

Validation activities should be planned in a validation plan, in an orderly manner. For instance, process validation should be performed after the various production and quality control equipment have been qualified, and not vice versa. Moreover, validation activities should be considered as an integral part of the quality assurance system, and should thus be documented in order to guarantee the necessary traceability. To this regard, the overall validation activities should be described in a general document, usually known as Validation Master Plan (VMP). VMP should include:

i) a general validation policy, with a description of the intended working methodology, and factors that may affect the quality of the intended radiopharmaceutical(s);
ii) a description of the facility, with a detailed description of the critical points;
iii) a description of the radiopharmaceutical preparation process(es);
iv) the list of production equipment to be qualified, including the extent of qualification required (e.g. IQ, OQ and PQ vs PQ only), and indications about equipment defined as critical (e.g. dispensing system working in a class A environment);
v) a list of the quality control equipment to be qualified, including the extent of qualification required;
vi) a list of other ancillary equipment, utilities, system and/or facilities to be qualified (e.g. Heating, Ventilation and Air Conditioning, or HVAC, or the gas distribution system), including the extent of qualification required;
vii) the general policy related to process validation;
viii) analytical methods to be validated; generally only those methods which are different from European Pharmacopoeia (Ph. Eur.) methods, for which only partial validation, performed by testing the most significant parameters (including e.g. system suitability test, detector linearity and LOQ), is usually required;
ix) cleaning validation of premises and equipment;
x) the general policy on re-validation, indicating frequency and conditions for re-validation;
xi) the personnel involved in the various activities, and a definition of responsibilities;
xii) a general change control and deviation policy, to be applied to all the involved protocols, aimed to specify how and when actions are required in case e.g. of test failures or an acceptance criteria is not met.

It is important to note that validation/qualification may represent a significant “burden”, in terms of the required time, personnel and financial resources, which are proportional to the complexity of the preparation process(es); this means that in case the facility is dedicated to the preparation of different radiopharmaceuticals, to be used for different clinical purposes, and multiple hot cells, automated systems and analytical equipment are used, an inadequate planning of validation activities may lead to an unnecessary workload and high costs. Thus, it is of paramount importance to clearly define in the VMP what has to be validated, the extent of validation required for each facility / system / equipment / analytical method, the actions to be taken in case of a significant change (e.g. the replacement of a production / quality control instrument with a different one) together with the conditions for re-validation / re-qualification. VMP should be periodically reviewed, especially in the light of the need for re-validation, and risk assessment methodology should be applied to take scientifically sound decisions.

A useful reference in the preparation of VMP is the document issued by PIC/S “Recommendation on Validation Master Plan….”(Validation Master Plan et al. 2017), but many other resources may be easily found on the web.

## Documentation

Validation/qualification activities should be documented. Validation/qualification protocols should include general information such as:

- A general statement on validation policy, with a description of working methodology and which validation stage is to be performed;
- a description of the instrument / process / step / analytical method to be validated;
- a list of the key personnel involved in the validation activities, including their individual training program and a clear definition of their responsibilities;
- in case of qualification of an instrument / equipment, a clear description of the test(s) to be performed (e.g. a pressure / temperature / radioactivity measurements), together with acceptance criteria;
- the reference equipment used during the above test (e.g. calibrated gauge, thermometer, dose calibrator, etc.), and related calibration certificate, if applicable;
- a reference to other relevant documentation (e.g. SOPs, instruction manuals, etc.);
- the raw data obtained during the execution of the experimental tests;
- a list of the deviations (if any) encountered during the execution of the protocol, together with a discussion about their possible impact on the considered instrument / process /operational step, and preventive / corrective actions, if applicable, which may provide useful suggestions to e.g. modify SOPs and operating protocols in general, prompt for possible equipment failures and allow for monitoring risks inherent to the intended systems /processes.
- a final summary, where results are commented, and a final statement about the intended process / instrument /method (the Validation Summary Report).

Validation protocols should be numbered following a logical order, using appropriate codes depending on the validation extent. For instance a possible code might be PQ-yyyy-nnn, where:

- PQ is acronym of Performance qualification
- yyyy indicates the year the protocol is performed
- nnn is a progressive number

Possibly the above information could be coded in a suitable SOP. At least the most significant information, such as test approval or rejection, as well as comments related to possible deviations, should be hand written.

In order to meet the necessary traceability, general quality assurance policy for documentation apply; for instance, type or hand writing errors should never be fully blurred or cancelled, but rather simply marked with a thick line, and updated information should be handwritten, dated and signed.

## User Requirement Specifications

**Fig. 1:**
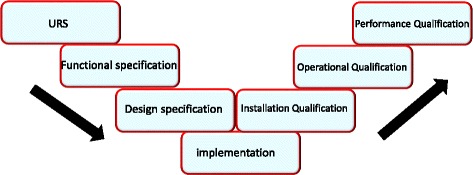
The V-shaped model

User Requirement Specifications (URS) may be considered as the first step in the validation “flowchart”, which can be summarized in the figure below (Fig. 1):

The aim of URS is to set parameters and related performance considered by the User as suitable to consider the system /equipment acceptable. URS should include:

- a description of the process / method to be carried out with the specific equipment (e.g. which kind of analytical procedures are to be performed with an HPLC system, the expected impurities, and the related limits, etc.);
- a detailed description of the intended instrument / equipment including computerized systems, if applicable;
- a brief description of the room / environment where the instrument / equipment is supposed to be installed;
- which utilities (e.g. gas supply, electricity, etc.) are required by the system / instrument, and which ones are available in the considered above room / environment;
- required performance of the instrument / equipment (e.g. eluent flow range for an HPLC pump, sensitivity for UV (ultraviolet) detector, etc.);
- documentation to be requested to the manufacturer (e.g. manuals, drawings, schematics, etc.);
- other useful information such as service and technical support.

A few examples may help to underline and clarify the above statement. An URS for an automated system for the preparation of RPs could include a list of significant parameters to be met by the system itself, such as radiochemical yield, preparation time and level of acceptable known impurities for a specific RP (e.g. [^18^F]FDG); general specifications could include the design of the system (e.g. cassette system vs re-usable systems), the purification process (e.g. semi-preparative HPLC vs SPE), the presence of “built-in” additional features (e.g. a sub-system for the automated performance of filter integrity test, etc.), and software specifications (e.g. the possibility to modify automated sequences that drive the preparation of RPs or the possibility to define software access and related privileges).

Following the same above described principles, a URS for a radio-HPLC could include specifications for sensitivity of radiochemical detectors, and/or accuracy, sensitivity, etc., for UV detectors, as well as specifications for software user access, audit trail policy, and more.

URS are of the utmost importance in case the intended system / equipment is not commercially available, and it has to be specifically designed. An example is represented by the Heating, Ventilation and Air Conditioning (HVAC) system, which is usually tailored to the needs of the User (e.g. air treatment units, as well as the size of the air conduits, will be chosen based on the requested level of “GMP” classification of the environments, the size and volume of the classified rooms, etc.), and whose design has to be specifically adapted to the local building layout. Another example could be the need to have custom made hot cells, specifically designed for non-standard research or production purposes, that may require additional shielding or larger internal working areas. In the above situations, URS are clearly to be considered as the first step in the “V-shaped” diagrams, and they are the basis for design qualification.

URS are also particularly useful in case of invitation to tender procedures, where they may represent the basis for tender official documentation, but they are generally considered as a useful reference document to define the intended use of the instrument and related acceptance criteria.

## Qualification of equipment

With the term “equipment”, it has to be intended all the instrumentation which is involved in the preparation and quality control of radiopharmaceuticals. Their functions, and general principles to be accounted for, will be described in the following two paragraphs, dedicated to the equipment for production and quality control, respectively. Although cyclotrons and nuclear reactors are, strictly speaking, directly involved in the preparation of an essential ingredient, the radionuclide, they will not be covered by the present guidelines, which is also in agreement with Annex 3 – GMP (EU et al. 2017a), that consider this important step in the preparation of RPs as a “non-GMP” step, and as such it’s not requested to be described and justified by the radiopharmaceutical manufacturers. There are practical reasons behind the above choice, that take into account the complexity and multi-tasking intrinsic nature of the radionuclide production equipment/infrastructures. More important, the quality of produced radionuclide(s) is carefully controlled, thus indirectly ensuring that the equipment is working properly and it is producing the intended radionuclide in proper amounts and quality.

Another general comment is related to the software systems, that are integral parts of most of the production and QC equipment, to date. They often play a critical role, performing the following tasks:

- instrumentation control (e.g. running a RP preparation or a dispending sequence in case of automated radiosynthesis or dispensing systems, regulating HPLC pump flowrate or handling the sample injections in case of radio-HPLC, etc.);
- acquisition of data coming from sensors / detectors;
- logging / storage of the acquired data;
- processing of the acquired data and creating reports;
- handle and assure traceability through audit trail functions;
- ensure safety and reliability of the preparations.

For the above reasons, a paragraph will be specifically dedicated to the validation of software and computerised systems, although reference will also be given when necessary throughout the discussion on validation of equipment.

Finally, qualification protocols are aimed to confirm that a system / equipment is properly installed, works correctly and leads to the expected results. This means that the successful outcome of a qualification protocol allows the equipment to be routinely used for the preparation / QC of radiopharmaceuticals, but does not eliminate the need for periodic testing of the instrumentation throughout their life cycle. The type of periodic tests, their recommended frequency and responsibilities are specific for each intended equipment, and they are usually part of the general quality assurance programmes, that should be in place in every radiopharmacy. Often they include tests already performed during the execution of qualification protocols, but that need to be periodically repeated to verify and ensure the correct functionality of the intended equipment. Although their detailed description is out of the scope of the present document, useful reference will be provided in the following paragraphs, especially (but not only) for the routine quality control testing of radioactivity detection and measurement instruments, such as dose calibrators, radio-HPLC “flow” detectors and gamma spectrometers.

### Qualification of production equipment

Equipment used in the preparation of RPs usually include: i) radiosynthesis system, which are often, but not necessarily, fully automated; ii) dispensing systems, which are often, but not necessarily, fully automated; iii) suitably shielded hot cells, where radiosynthesis and dispensing systems are located, for radiation protection purposes; telepliers and manipulators are sometime used in those systems not equipped with fully automated devices; iv) hot cells/isolators for manual preparation of RPs (e.g. these are frequently used in the preparation of Tc-99 m labelled kits or in cell labelling); v) dose calibrators. Other instruments or accessories may be used, but they will not be considered in detail by the present guidelines. On the other hand, the same principles and methodologies that will be described for the typical equipment also apply to less frequently used instruments. It has to be considered that production equipment complexity range from relatively simple instruments, such as dose calibrators, to more complicated devices such as automated systems for radiosynthesis or dispensing. Qualification activities should be focused on the most critical components, evaluating the possible effect of failure or miscalibration on the general performance of the system and, in turn, on the quality and safety of the desired RP products.

Installation of production equipment may be preceded by additional evaluation steps, namely Factory Acceptance Testing (FAT) and Site Acceptance Testing (SAT). FAT/SAT are particularly useful in case of complex and/or bulky equipment, or when the intended instrument has been specifically designed, based on URS. A good example is represented by hot cells, whether or not they include automated systems. Hot cells are indeed bulky, and sometimes require some degree of customization, especially in case they have to be used for non-standard processes. During FAT, functionality of major components (buttons, fan, filters, etc.) may be tested, as well as parameters typically verified during OQ such as air velocity, leak tightness or particle contamination. The above tests might help to reveal possible malfunctions or deviations, that may be fixed directly at the Factory, before the shipment. FAT are usually repeated on Site (SAT), and they can be considered as part of the whole qualification.

#### Radiosynthesis system

“Initial qualification and periodic qualification should be planned in the master document describing each automated module. Initial qualification should include IQ, OQ and PQ. IQ should include the verification of the designed module specifications, the check of installed instrumentation and the integration of working and maintenance instructions in the master document of the module. The functionalities of the automated module without reagents nor chemical components should be checked during OQ, which should also include: i) a verification of the software user access policy, with reference to the different possible level of privileges (e.g. administrators usually have the right to modify any parameters, sequences, methods, etc., while operators should have the possibility to run dispensing programs only); ii) a verification of the software sequences, if applicable; iii) a verification of the possible effects of a general power failure (e.g. to check for the presence and / or the need for an UPS; iv) a verification of the calibration status of the major components; v) a verification of data backup and restore. If the module is a commercial one, the user should ask the supplier to perform a qualification according to internal procedures or to propose a procedure to be performed by the user. If the module is custom made, the user should check that all functionalities, defined in the URS document, meet the specifications included in the master document describing the module. This should include the movement of actuators and the calibration status of the probes (temperature, pressure, and radioactivity). PQ of the module should be conducted by performing three complete runs of a representative process covering all normal operations for the concerned preparation process. For example, a module including a preparative chromatographic system should be qualified selecting a RP preparation process which includes a chromatographic purification. PQ should demonstrate that the module is suitable for the intended application in real conditions of use.

Each automated module should follow a programme of periodic qualifications of the probes (temperature, pressure, and radioactivity) in order to re-calibrate them if needed. For major updates or repairs of the mechanical part, or in case of major modifications of the control software, a risk assessment should be performed in order to evaluate the potential impact on the process performed with the module. OQ and PQ should eventually be performed as conclusions of the risk assessment” (Aerts et al. 2014).

#### Dispensing systems

General principles outlined for radiosynthesis devices also apply for dispensing systems, which allows for vial or syringe dispensing starting from a “mother” solution prepared using the automated radiosynthesis module. IQ should include: i) a verification of the documentation, such as instruction manuals, drawings, electrical / pneumatic schematics, specifications, certificates; ii) a verification of the major installed components, such as valves, tubing, software, programmable logic controller (PLC) or other suitable control, pumps, balances, control panels, PC, software version, etc. Verification is aimed to check that components are installed in a proper way, by comparison with URS and / or the documentation provided by the manufacturer, if applicable; iii) interconnections between the major components (e.g. cables, tubing assemblies, etc.); iv) a general check of the electrical connections and gas supply piping.

OQ should consider: i) a verification of the software user access policy, with reference to the different possible level of privileges (e.g. administrators usually have the right to modify any parameters, sequences, methods, etc., while operators should have the possibility to run dispensing programs only); ii) a verification of the software sequences, if applicable; iii) a verification of the possible effects of a general power failure (e.g. to check for the presence and / or the need for an UPS; iv) a verification of the calibration status of the major components; for instance, in several dispensing systems, vial filling accuracy is based on balances that weigh the solution during filling operations; balance is in this case a critical component and its performance could be evaluated during OQ by comparison with a calibrated precision balance, using certified weights. Certificate of calibration of the reference balance and weights should not be expired and should be included in the validation documentation. Dispensing systems for individual syringes preparation are preferably based on direct radioactivity determination using dose calibrators: in this case the dose calibrator is the critical component, whose calibration status need to be verified during OQ (see below). One more example of critical components in dispensing systems are the pumps often used to draw / push fluids through tubing assemblies; again, a verification of their calibration (e.g. by measuring dispensed volumes with a reference precision balance) should be performed during OQ; v) a verification of data backup and restore.

PQ of dispensing systems might be carried out by performing at least three successful dispensing cycles in typical working conditions, i.e. using radioactive solutions of the intended activities and radioactive concentrations, dispensed in a representative number of vials / syringes.

#### Shielded hot cells

Hot cells may be used to accommodate automated or remotely controlled radiosynthesis apparatus or, more simply, to provide the operators a suitable environment to prepare RPs, manually or with the help of tele-pliers, their major functions being to protect the operators from radiation burden (useful calculators to determine the required shielding thickness may be found on the web, see e.g. (Radprocalculator)), and to guarantee an environment with suitable air quality and cleanliness, which is critical for the microbiological quality of the products. Generally, working area is tightly sealed, and a negative pressure is operating, to allow potential radioactive exhaust to be collected to safe containment systems, such as shielded gas cylinders or retardation pipes. Qualification extent for hot cells is dependent on their complexity, that may range from a simple working surface surrounded by an adequate lead shielding, to fully automated dispensing system which are embedded and integrated in the hot cell whole structure. However, there are common characteristics that may allow to set general principles for their validation.

IQ follows the same general concept above depicted for automated systems, and basically consists of a series of verification of the documentation, the major installed components and their interconnections. Specific test for OQ might consider:

i) a leak test, to verify the tightness of the working area with respect for the external environment; the test may be performed by simply measuring leak rate after negative pressure has been brought to its maximum, and ventilation / extraction have been switched off, thus isolating the hot cell itself;
ii) an air velocity test, to determine the suitability of ventilation above the working area, where RP preparation and dispensing operations take place; an alternative test may be the measurement of air particle contamination, using portable or stand-alone calibrated particle counter devices, which provide and indirect, but nonetheless effective, measure of air quality; indeed, class B or class A environment, as defined by EU GMP – Annex 1 (EU et al. 2017b), are often claimed by hot cell manufacturer. In OQ, these test are performed “at rest”, with working area in normal operating conditions, but without personnel intervention.
iii) hot cells doors are usually interlocked for safety reasons; for instance, in case of hot cells used for the preparation of PET RPs, radionuclide transfer from the cyclotron is not allowed if hot cell doors are open; other common safety interlocks link radiation levels inside the working area with hot cell door opening, which is not allowed in case the level is above a defined threshold. Test to verify functionality of interlocks are typical operations to be included in OQ protocols.
iv) Other tests, such as laminar flow pattern determination (e.g. using a smoke trace), may be performed, depending on the hot cell characteristics. It has to be underlined once again the need to focus on critical parameters.

It may be appropriate to consider PQ of hot cells in conjunction with OQ, as there is no significant difference in their mode of operation during the preparation of the RPs or at rest. On the other hand, this is not true in case of manual or semi-automated operations, when manipulations may affect laminar flow pattern, e.g. due to the movement of the operating personnel arms through the gloves. Thus, the above test should be executed both at rest (OQ) and “in operation” (PQ). As for particle monitoring, it has to be noted that radioactivity may strongly influence the instrument response, as radiation pulses may be erroneously “counted” by the particle monitoring system, and thus particle contamination may be overestimated. Keeping this in mind, for hot cells dedicated to automated preparation or dispensing operations, PQ tests should be performed during their normal work cycles (e.g. during the preparation or the dispensing of the intended radiopharmaceuticals). In case of hot cells used for manual RP preparations, which are typically performed by personnel through suitable sealed gloves, PQ tests should be performed during normal workflow (e.g. during the preparation of Tc-99 m labelled kits).

#### Dose calibrators

IQ for dose calibrators should be straightforward, considering that these kind of instruments are relatively simple and consist of a suitable detector connected to a PC or to an electronic control case with a display. Thus, a check of documentation and installation conditions (electrical connections, cabling between detector and measuring / display device) should be sufficient. OQ test should be aimed to verify calibration status of the dose calibrator. This could be done with accuracy and reproducibility tests, to be performed using suitable calibrated radioactivity sources. Chosen radionuclides should be of adequate activity, energy and half-life (e.g. Cs-137,with γ emission at 662 keV, whose T_1/2_ is = 30 years). OQ might also include other tests, such as verification of calibration through accurate measurements of the output current in response to increasing activities, but usually the monitoring of the above cited parameters is considered sufficient to provide an adequate operational qualification of the instrument. The same test should be performed with the aim of qualifying the performance of the instruments, but using the intended (or one of the intended, in case more radionuclides are measured with the same instrument) radionuclide(s). For instance, in a [^18^F]FDG preparation facility, accuracy, reproducibility and linearity should be determined using F-18 with activity in the normal working range. For PQ purposes, also Limit of Quantitation (LOQ) should be determined. OQ and PQ tests should take into account the geometry of the sample (e.g. shape and size of the container, and distance to the sensitive surface of the detector). Re-qualification policy of dose calibrators should account that daily checks (e.g. constancy tests) are usually performed, and also verification of linearity and reproducibility are relatively frequent, so as to avoid the need of re-qualification, that should be only done in case the instrument is moved to a different location or due to other significant changes. There are a number of useful reference documents that may help during the implementation of the IQ, OQ and PQ validation steps. Table 6 of EANM guidelines on “Acceptance testing for nuclear medicine instrumentation” (EANM guidelines) provide a list of tests to be performed both at the acceptance of the instrument and to periodically verify its correct functionality. More experimental details related to the above suggested tests are described in EANM guidelines on “Routine quality control recommendations for nuclear medicine instrumentation” (EANM guidelines). Finally, recommendations relevant to assuring the continuing acceptability of the performance of radionuclide calibrators are set by European Commission Radiation Protection document n° 162 “Criteria for Acceptability of Medical Radiological Equipment used in Diagnostic Radiology, Nuclear Medicine and Radiotherapy” (EU Commission & Radiation Protection n. 162).

### Qualification of QC instrumentation

Quality control activities may range in complexity from relatively simple test with TLC, which is often the main QC test required in case of Tc-99m labelled kits, to a full series of QC tests, including HPLC, TLC, GC, gamma spectrometry, etc., in case of PET radiopharmaceutical preparations. QC equipment may include instruments normally used in analytical chemistry, such as pH meters, analytical balances, HPLC or GC, and instruments specifically designed for the analysis of radioactive samples, such as activity detectors conjointly used with HPLC or TLC, and gamma spectrometers. Moreover, the need to control microbiological contamination of injectable radiopharmaceutical preparations make devices designed to monitor endotoxin levels familiar to the radiopharmacists.

IQ for QC equipment follows the general rules already depicted for production equipment, and verification of the documentation, drawings, schematics, cables and piping, a general check on SOP, logbook, etc., and verification of environmental conditions and utilities here also apply. OQ and PQ are more specific for the various instruments, and will be described with more details. It has to be underlined once again that IQ, and also OQ, may be also be performed in close cooperation with the instrumentation manufacturer, thus allowing to reduce workload for local radiopharmacy staff.

### Radio-HPLC

A radio-HPLC system is typically composed of a pump, which drives the eluent through the various detectors and columns, the detectors themselves, one of which is always a radioactivity detector, while the others are needed to identify and quantify non-radioactive species, and their selection is depending on the intended application. The most frequently used detectors are UV detectors, but conductivity or electrochemical (or others) detectors are also used for specific applications. These detectors will be hereinafter defined as “mass detectors”. Injection of the sample may be performed manually or automatically, by means of an autosampler. Chromatographic columns may be kept at room temperature or heated, by means of a column oven. Finally, most of the HPLC systems currently available are controlled via a suitable software, which is also used to acquire and process signals coming from detectors. From a validation perspective, HPLC may be considered as a sum of different components that may be tested individually. Thus, OQ and PQ test should be designed specifically for e.g. UV detectors, as well as for radiochemical detectors, while control and acquisition software may be evaluated as a whole. OQ on radiochemical detectors may include a linearity verification of the voltage output, in response to decreasing level of radioactivity. A sample of the intended radionuclide/radiopharmaceutical is suitable for this purpose. OQ test on UV detectors usually include: i) test on wavelength accuracy, using a suitable known reference standard; ii) noise and drift test, which can be performed running flow for a suitable time (e.g. 60 min) and recording and allowing software to record the above parameters (some instruments may already have software routines designed to run the tests); iii) a verification of absorbance accuracy using reference standard, which can be easily purchased from commercial supplier, iv) test on software user access and related privileges. Similarly, other “mass detectors” such as conductivity detectors might be OQ checked for linearity and reproducibility using standard ionic solution (e.g. chlorides, sulphates, etc.). HPLC pump may be tested for accuracy and precision by collecting and weighing, using a calibrated analytical balance, a statistically significant number of samples (e.g. 10 samples, collected at a flowrate of 1 ml/min). Column oven, if present, should be checked for its capability to maintain the selected temperature, by setting a range and measuring, using a calibrated thermometer, a range of temperatures. Similarly, accuracy, precision and linearity test might be performed on the autosampler, with the aim to verify their capability to reliably inject samples of the desired volumes. Irrespective of the way the samples are injected (manual or automated), the injection system needs to be cleaned between injections: carry-over is another typical OQ test, aimed to prove the efficacy of the cleaning procedure. Carry-over should be tested by repeatedly analysing samples of mobile phase following the injection of samples containing significant amounts of the intended analytes; to verify carry-over of UV or other “mass detectors”, samples should be taken from the higher concentration solution used in linearity test; for radiation protection purposes, carry-over tests on radiochemicals should be avoided, and the results obtained with test on mass detectors should be considered as sufficient to demonstrate the cleaning efficacy.

PQ protocols on mass detectors should include tests to verify precision (or reproducibility) of the detector output, by injecting at least 5 samples of the intended “cold” counterpart of the desired radiopharmaceuticals (e.g. [^19^F]FDG for [^18^F]FDG, or [^12^C]methionine for [^11^C]methionine) at a concentration included in the typical working range; also linearity test, using at least 5 different solutions with increasing concentration in the normal working range, should be performed. PQ test on radiochemical detectors should be aimed to check precision and linearity as well. However, due to radioactive decay, a single sample of suitable activity might be used, and area values obtained from the related chromatograms should be recalculated using the decay law (A = A_0_e^-λt^). This PQ tests could be considered part of method validation, which will be the subject of a dedicated guideline.

### Gas chromatography

In the field of radiopharmacy, gas chromatography is very often (although not always) used for the analysis of residual solvents. The instrument is made of a column (often a capillary column) placed in an oven, through which a carrier gas is swept; the most popular detector is Flame Ionization Detector (FID), which is suitable for residual solvents analysis, but other types may be used, depending on the selected application. Similarly to HPLC, sample injection system may be manual or, more frequently, automated (e.g. head space injection system). IQ protocols don’t differ significantly from those already described for radio-HPLC. OQ tests on FID detectors should include sensitivity. Head space injection system, if present, should be tested for precision and accuracy using reference standard, in order to verify its capability to reliably inject the selected volumes, and for temperature, using a calibrated thermocouple. A leak test, to check the tightness of the injection system, has also to be performed. Finally, test on carry over within the injection system is also recommended. Oven temperature is another critical parameter that should be checked during OQ, by means of a calibrated thermometer; a series of measurements allows for accuracy and precision determination. Also carrier gas flowmeter should be checked, by comparison with a calibrated flowmeter. PQ, as usual, helps to demonstrate that the system is capable to yield the expected performance in normal operating conditions. Precision and linearity should be checked using a reference solution of one or more of the analytes that are expected to be quantified during normal QC operations (e.g. acetonitrile, ethanol), while for linearity determination, a series of solutions with increasing concentrations of the interested analytes should be prepared and analysed. The same data obtained following the above tests, could then be used for the validation of analytical methods.

### Radio-TLC

Radio-TLC scanners are mainly used to determine radiochemical purity of radiopharmaceutical preparations. Radio-TLC are often scanners that drive a TLC sheet or plate under a suitable sensor capable to detect radioactivity. Autoradiography systems may also be used for this purpose, that take advantage of the capability of a suitable phosphor plate to store the radioactive signal and release it in the form of a suitable luminescence, and that may thus create a kind of “latent” image of the spots generated during the TLC run by the separation of the analytes. IQ follows the same principles already depicted for other analytical instruments. OQ and PQ may be considered conjointly, and usually tests on reproducibility and linearity, using a solution of the desired radionuclide with suitable activity range should be performed. Reproducibility may be evaluated by deposition, using preferably a calibrated micro-pipette, of a few microliters of the radioactive solution in different position of the TLC plate. During data acquisition and calculations, decay should be accounted for, especially in case of very short half-life radionuclides. For linearity purposes, a single spot could be deposited and acquired at suitable user defined intervals. Other OQ tests may be related, as usual, to the software system, by checking software access policy and privileges, and archiving/backup functions.

### Gamma spectrometer

Gamma spectrometers are used both for identification and radionuclidic purity determination purposes. Most common detectors are HPGe (high purity germanium), which are to be preferred due to higher energy resolution, and thallium activated NaI detectors, that have better sensitivity but much lower energy resolution. Detectors are suitably shielded, to reduce background, and connected through a cable to a PC equipped with a proper software. HPGe also need to be cooled, in order to reduce thermally induced leakage current, and effectiveness of the cooling system, irrespective if this is done via liquid nitrogen or electrically, has to be carefully controlled during normal operations and during qualification tests as well. With such a simple configuration, IQ is also quite simple, and it may be performed, as already described for other instruments, by a general check of the documentation (order, manuals, other documents provided by the supplier, logbook, dedicated SOPs, installed software, versions, etc.). OQ may include an energy calibration of the instrument, with the aim to verify that detected energies match with expected values. Both mono- and multinuclide calibration sources may be used. Multinuclide or single-nuclide multi-energy (e.g. Eu-152) sources should be preferred, so as to check calibration status in a broader energy range. Usually, typical working range of the above instruments is indeed 0–2000 KeV. A minimum of 6 acquisitions for each of the selected energy signals, followed by coefficient of variation (CV%) calculation allow for energy calibration determination. Efficiency is another parameter to be considered in OQ, especially when gamma spectrometry is used for quantification purposes. Here also multinuclide sources are ideally suited, as they allow for quantification of radioactivity amount of the various nuclides, provided that they are sufficiently long lived (medium half-life radionuclides might also be used, but errors are higher). PQ is depending on the intended use of the instrument, but it generally includes reproducibility and linearity tests, to be performed with the radionuclides expected in the RP preparation of concern. The sensitivity of an instrument is usually measured, as already described above, using calibrated standards at the proper concentration. In case of gamma spectrometer, sensitivity may be expressed by a parameter known as Minimum Detectable Activity (MDA), which may be considered similar to the Limit of Detection (LOD), and which is dependent on many factors (background, geometry, etc.) and it may vary from run to run for the same radionuclide. Thus, although MDA might be determined, for example, during OQ test with calibrated source(s) or during PQ with the intended radionuclide, it would make more sense to evaluate it during validation of the specific analytical method. It is also important to establish the maximum detectable activity range, as the saturation of the detector may lead to underestimation of the radioactivity.

## Validation of analytical methods

Quality Control of radiopharmaceuticals, for which dedicated monographs of Ph. Eur. are available, are straightforward, as the monographs define which controls have to be performed, including the related method of analysis with experimental details (e.g. stationary phase, mobile phase, flowrate, wavelength in case of HPLC analysis with UV detector, etc.) and acceptance criteria. Following the above specifications, the analytical methods of concern do not need to be validated, but rather verified in each individual laboratory through the execution of tests aimed to verify that the method has been implemented properly (e.g. system suitability test, detector linearity and LOQ). In case a monograph for the intended RP is not published, or in case the monograph exists but for any reasons it is preferred to use a different method, its suitability need to be assessed and demonstrated through a validation procedure. Guidelines for validation of analytical methods have been released by ICH (ICH guideline Q2(R1) Validation of analytical procedure: text and methodology), which provide general information and guidance about the parameters to be tested (e.g. accuracy, precision, linearity, etc.), how to test them and when; for instance, the above guidelines state that the determination of repeatability should be performed after a minimum of 9 analyses, covering the specified range of the procedure, etc.

During validation of analytical procedure three common analytical procedures may be recognized:

- Identification test, which is aimed to contribute to the identification of the desired product or other analytes in the sample. In case of RPs, identification of the intended RP is often carried out exploiting the two distinct characteristics of any RP: i) the “pharmaceutical” part is identified through the chromatographic comparison of the retention time of the main radioactive peak with retention time of the “cold” standard (e.g. [^18^F]FDG is identified through the analysis of a [^19^F]FDG sample); ii) the radionuclide part contributes to the overall identification of the RP thanks to the gamma spectrometry analysis of the emitted energies (e.g. in case Tc-99m is the intended radionuclide, an emission at 140 KeV is expected).
- Testing for impurities, which can be quantitative or limit tests. In case the impurities are well characterized (e.g. the concerned impurity is the chemical precursor of the radiolabelling), testing may be feasible, and rules set by ICH guidelines apply.
- Assay test, that are performed to quantitatively measure the analyte of interest (usually, the desired RPs).

An analytical method should be re-validated in case of changes in the RP preparation process that may affect the quality of the final products, when purification components are replaced by different ones (e.g. alumina cartridges are replaced by ion exchange cartridges) or the purification method is changed (e.g. HPLC vs SPE) and in other circumstances.

Analytical methods used for the QC and characterization of RPs are sometimes typical analytical methods (for example, analysis of residual solvents using GC); in these cases, ICH guidelines apply without significant adaptations. On the other hand, specific adjustments are required in case of radioanalytical methods, such as radio-HPLC, radio-TLC and gamma spectrometry, and they would need to be considered with more details. For this reason, and in consideration of the wide variety of possible application in the field of radiopharmaceutical preparations, validation of analytical methods will be the subject of a dedicated document. Moreover, practical examples of validation of analytical methods of routinely used RPs may be found in the EANM guidelines on the preparation of IMPD (Todde et al. 2014).

## Computerised systems

As already mentioned earlier, computerized systems are ubiquitously used and most of the instrumentation of concern in the field of radiopharmacy are controlled by a wide variety of hardware / software systems. Thus, validation of software should be considered as an integral part of the general validation policy (http://ec.europa.eu/health/files/eudralex/vol-4/annex11_01-2011_en.pdf. Accessed 31 Mar 2017). Two different general approaches are possible: i) validation / qualification of a production / QC instrument as a whole (holistic approach), in which the computerised system is considered as a part, although significant, of the whole instrument, and validation of hardware / software is thus performed consistently; ii) validation of computerised system as an independent entity. Whatever is the chosen route, the following principles apply:

- requested characteristics of the software / hardware should be described in URS;
- data safety should be ensured, so as to minimize the risk of loss of data or wrong data entry by the operators;
- initial configuration of the systems should be performed by qualified personnel;
- as already stated previously, access to the software should be allowed for authorized persons only, and it should be regulated by means of appropriate login / password, and the allowed operations should be different, depending on the various functions;
- backup functions should always be enabled, and the way, the chosen storage medium (e.g. stand-alone devices such as USB pen drives, CD rom, external hard disk, or network backup disks) and backup frequency should be defined through appropriate SOPs; restore functions of the backed up data should be verified, as well;
- printout function of the stored data should be checked;
- audit trails functions should be considered mandatory, in order to have the necessary traceability of the operations performed using the interested software; for instance, information about access (who and when accessed the software), changes (e.g. about deleted files, change to operating methods, new methods, etc.), software / hardware updates should be automatically recorded by the software; in case the audit trail is not enabled, alternative procedures to ensure operation traceability should be put in place (e.g. printing and / or recording information about performed operations on dedicated logbooks);
- the risk related to possible accidental loss of data or software functionality should be carefully evaluated, and executable copy of the interested software should be available and fully compatible with the hardware equipment;

A useful reference while validating computerised systems is the PIC/S guidance on “good practices for computerised systems in regulated “GXP” environments”(PIC/S Guidance), whose main goal is to help users in understanding requirements and the level of validation to be performed and, which is even more important, to help suppliers in developing their systems complying with general rules of good practice. It means that whenever the purchased systems have been developed complying with GAMP, validation extent required to the end user is minimized. Moreover, supplier should provide appropriate documentation.

IQ for a computerised system might include the following controls:

- check purchase order, shipment documentation, packaging, etc.;
- the software has been loaded correctly;
- power supply and electrical protection are suitable for the intended applications;
- PC and hardware have been properly connected;
- the system is working properly after power up;
- collect information about software / hardware / operating system versions, date and place of installation;
- include a brief description of the room / site where the systems have been installed;
- software / hardware certification are available (if any).

OQ would be more focused on a functional verification of the software / hardware, and might consider the following verifications:

- check on data integrity, accuracy, security and reliability;
- verification of operating sequences, such as automated synthesis module routines used to perform part of the preparation process (e.g. a purification operation, or fluid transfers through different parts of the modules, etc.) or acquisition sequences for an analytical instrument;
- a verification that different login/password credentials for access are working and lead to different operating privileges;
- verify software / hardware safety, e.g. through breaking up o f power supply;
- verify data safety, by means of attempts to change, delete, copy/paste, etc.

Finally, PQ on computerised systems might include the following tests:

- test specific SOPs, dedicated to the intended RP preparation process, for use and maintenance of the computerised system;
- as PQ is typically aimed to verify that the system is capable to properly perform the tasks for which it has been purchased / built, PQ for computerised systems tests could be merged with general PQ of the intended instrument / system / utility. Thus, please refer to the information provided in the relevant section for e.g. PQ on automated synthesis systems, dispensing systems or for analytical instrumentation

## Validation of classified rooms

Preparation of parenteral injectable solutions requires special care in the manipulation of the starting materials /intermediates / finished products, that may potentially be subject to microbiological contamination in the form of bacterial endotoxins and vital microorganisms such as bacteria and fungi. To this regard, Annex 1 – GMP (EU et al. 2017b) set general guidance about technical characteristics of classified environment, as well as of the tests to be performed together with related acceptance criteria for particle and microbiological contaminations. The possibility to establish and maintain a classified environment depends on several factors, such as the technical specification of HVAC system, construction details of the premises, characteristics of equipment, dressing and behavioural rules for the operating personnel, cleaning and sanitization procedures, sterilization, etc. Qualification of classified environments is challenging for typical radiopharmacies, as it requires skills and instrumentation which are often not available. Moreover, differently than the above described production and QC instrumentation, which are usually commercially available, even DQ plays here a crucial role, as rooms and HVAC are specifically designed for the intended use, and their characteristics may significantly affect day-by-day operations and general compliance with EU guidelines. DQ will have to be performed in tight connection with URS requirements, and will have the goal to verify that e.g. requested utility services are available and suited for the intended purpose or that the systems will be easy to be calibrated and maintained and may operate in a manner safe for the products and for the operating personnel. IQ of HVAC include a careful verification of all the installed components, to check that e.g. valves, pipes, shutters, ventilation machines are properly installed compared with project layout, and that they are properly labelled. Of course a general check on documentation (drawings, layout, component specification, list of the suppliers, operating manuals, etc.) is here of paramount importance. OQ of HVAC, which plays a critical role in determining the quality of air, usually foresee tests on air flowrate, HEPA filters integrity, the number of air exchange / hour, particle and microbiological contamination. For these reasons, full qualification of classified environments is usually sub-contracted to suitable specialized service companies. However, the following tests, that can be considered as representative of the general classification status of the intended rooms, could be performed, provided that at least an air particle counter and an incubator are available.

1) the effect of lack of power supply on HVAC efficiency; this test may be easily performed by turning off and on the general power supply, and checking whether the main functions are correctly recovered or not;

2) particle contamination test, that has to be carried out using a suitable calibrated instrument, following general methodology set by ISO standard (EN ISO 14644 1). Number of air samples may be determined using the following formula:

$$ N L=\sqrt{A} $$

Where “NL” is the number of samples to be taken, and “A” is the surface of the classified area (expressed in m^2^); a minimum of two samples should be considered, notwithstanding the surface area. In case of class “A”, it is necessary to collect at least 1 m^3^ of air / point (EN ISO 14644 1), while for other classes, 1 m^3^ should be the total volume for the considered whole room /environment. The following general formula may be used to calculate the minimum sampled volume:

$$ V s=\frac{20}{Cn, m}\times 1000 $$

Where:

- Vs is the minimum single sample volume per location, expressed in litres
- Cn, m is the class limit (number of particles / m^3^) for the largest considered particle size specified for the relevant class
- 20 is the defined number of samples that could be counted if the particle concentration were at the class limit

Test should be done in “at rest” conditions, as defined by Annex 1 – GMP (EU et al. 2017b).

3) decay / recovery test, which is intended to determine the time needed to recover the specified class after e.g. HVAC is intentionally switched off for a defined time.

4) clean-up test; in principle, this test is aimed to determine the time required to switch from one condition to another; in case of cleanroom, that may be represented by the time it takes to “clean-up” from “in operation” to “at rest” conditions, and can be experimentally measured monitoring appropriate parameters, such as airborne contamination.

PQ may be performed by: i) repeating the particle contamination test in “in operation conditions”, which means with personnel normally operating in the lab; ii) verification of the microbiological contamination of the air and surfaces, the latter being checked by means of agar contact plates filled with a suitable media, and the former using agar settle plates; number of plates and their position have to be chosen with a rationale based on the expected microbiological risk; to this regard, contact plates should be scratched on representative positions on the floor, walls and major instrumentation (inside/outside hot cells, external surface of automated system, workbench, etc.), while settle plates should be located more or less in the same points above cited. Agar plates should then incubated at 25 ± 2 °C for three days, and at 35 ± 2 °C for the next 2 days. Acceptance criteria are depending of the desired classification level and are set, as usual, by Annex 1 – GMP(EU et al. 2017b).

## Cleaning validation

Cleaning validation is aimed to verify the effectiveness of a cleaning procedure. Two general cleaning procedures are of concern in the preparation of RPs : i) cleaning of production/dispensing apparatus, with special emphasis for those parts of the equipment which come into contact with reagents /solvents /intermediates / finished products; ii) cleaning of the external surfaces of the equipment (e.g. hot cells, laminar flow cabinet, workbench, furniture, etc.). Considered that aim, procedures, and critical steps are different, they will be described separately.

### Validation of the procedure for the cleaning of production equipment internal surfaces

Production of RPs is often performed using automated or at least remotely controlled devices. A useful guidance, edited under the umbrella of EANM Radiopharmacy Committee, for the use, installation, cleaning, and validation of automated systems has been recently published (Aerts et al. 2014), and general principles of cleaning validation may be found. In general, automated systems may be of two distinct types, depending on the nature of the so called “chemistry part” of the system, which is defined as “*an interconnected network of containers in which gaseous, liquid and/or solid reagents and components can be moved, mixed and/or transformed to obtain the desired final product*”(Aerts et al. 2014). With “cassette” systems, the chemistry part is disposable, and replaced every time a new preparation begins, while in non-disposable systems the chemistry part may potentially be re-used for an undefined number of times. In the latter case cleaning operations and, in turn, cleaning validation are clearly more critical than in the former. “*Validation of the cleaning processes should be performed prior to the use of the automated module, to demonstrate that cleaning operations are efficient to fulfil the established specifications in the area of effective operation*”(Aerts et al. 2014). A thorough knowledge of the chemistry involved in the preparation process is required, so as to identify the possible impurities left over inside the chemistry part surfaces, select proper limits and acceptance criteria of carry over and, which is of the utmost importance, design a suitable cleaning process. Cleaning validation need to be performed both in case the automated system is used to produce a single radiopharmaceutical (e.g. [^18^F]FDG) and in case it is used to prepare different RPs, which may pose additional problems of cross contamination. Cleaning validation should include at least three productions of the desired radiopharmaceutical, followed by three cleaning procedures. The latter should be designed with the aim to keep carry over at a minimum extent. For validation purposes, cleaning steps should be followed by a careful sweeping of the inner surfaces of the chemistry part with a suitable (aqueous or organic, or both) media, capable to solubilize most of the residuals of impurities. The above operations should be designed so as to ensure that all the possible surfaces that get in contact with reagents / intermediates / final product are suitably swept by the above media. Washing solutions should then be collected, and samples submitted to quality control procedures. Analytical methods should be sufficiently sensitive to detect the established acceptable level of the residue or contaminant. The above “sweeping” step should keep out multiple use chromatographic support, such as liquid chromatography columns, due to their inherent characteristics and capability to retain impurities. In case the automated system is used to produce different RPs, cleaning validation protocols should demonstrate that cleaning procedures are effective irrespective of the order that the various RPs are produced.

As already stated above, cleaning validation protocols are less critical in case single-use, disposable systems are used. This general consideration apply to both “cassette” automated modules for RP production, and to dispensing systems used to prepare syringes with individual patient doses or multi-dose vials. However, should the above systems include non-disposable parts (e.g. typically the radionuclide transfer line from the cyclotron), the same principles previously depicted apply to the parts of concern.

The same considerations apply in case of microbiological contamination, which is less critical in case of “cassette” systems, due to their single-use characteristics. Moreover, some commercially available kits are sterile. In case of non-disposable system, bioburden is the method of choice to validate cleaning procedures. Typically, three preparation runs are performed using the same conditions set for normal routine preparations, but without using radioactivity and avoiding final sterilization (e.g. in case the RP solution has to be sterilized by filtration, filter is not included in the preparations dedicated to bioburden testing). As ionizing radiations, depending on the amount and radiation pattern of the starting radionuclide, may play a role in keeping the microbial populations low, the lack of radioactivity during the simulation of the preparation procedure may be considered as a worst case scenario. The three simulated preparation runs yield solutions, which are then analysed following routine procedures for bioburden test. Typical acceptance criteria is 10 Colony Forming Unit (CFU) / 100 ml (Note for guidance on manufacture of the finished dosage).

The choice of the cleaning media, which may be a solvent (e.g. acetone, ethanol, isopropanol, etc.), a gas (e.g. ozone, ethylene oxide), or a combination of different solvents/gases, is clearly very important, as they should be: i) effective in the removal of both chemical and microbiological impurities, ii) easy to be removed from the system after the end of the cleaning procedures, iii) non-toxic, iv) inert with respect for the interested internal surfaces, and v) they should not yield additional impurities. For instance, acetone is suitable in solubilizing chemical impurities, due to its polar characteristics, and it’s easy to be removed, due to its low boiling point, but it is not very effective with microbiological impurities, and ethanol, isopropyl alcohol or a mixture of the above solvents might be preferable.

### Validation of the procedure for cleaning external surfaces

External surfaces may include laboratory floors and walls or workbenches, as well as internal and external surfaces of the hot cells / laminar flow cabinets, and external surface of the preparation equipment. They should be cleaned following written procedures, that set which detergents / disinfectants / biocides have to be used, the cleaning operation frequencies (e.g. daily, weekly, etc.), and turnover policy on biocides. Chemical, radiochemical and radionuclidic residues are usually not of concern for contact surfaces, as their presence should be considered as a consequence of inadvertent contamination. Further, RPs are generally prepared in small scale, and low amount of reagents / solvents are used, which further decrease the risk of “chemical” contamination e.g. on workbenches or around the automated systems surface. The small scale “size” of RPs preparations has also to be considered in view of a risk evaluation due to the operating personnel, which is usually low in number and occupancy factor. Thus, validation of cleaning of contact surfaces is mostly aimed to demonstrate that microbiological contamination is kept within the proper limits, depending on the desired classification level (EU et al. 2017b). Such a cleaning validation protocol should include:

- a description of the facility, including the requested level of room classification;
- a description of the instrumentation / equipment installed in the classified rooms, and their locations;
- the cleaning procedures (also through a reference to the dedicated SOPs);
- a description of the cleaning products (detergents, disinfectants, biocides);
- a description of the process(es) carried out in the interested rooms, with special care in case of “multitracer” production in the same environments;
- the sampling procedures (e.g. direct surface sampling using swabs or contact plated petri dishes, etc.);
- the sampling campaign and criteria; sampling points considered to be most critical (e.g. hot cell working surface, the laboratory area where personnel spend most of the time, etc.) should be discussed and justified;
- test methodology, including the media used to collect samples (e.g. contact plates, air sampling instrumentation, type of microorganism growing media, etc.);
- acceptance criteria;
- protocol discussion and conclusion.

The design of a cleaning validation protocols might take advantage of risk analysis based on the knowledge of the intended RP preparation processes and of the established cleaning procedures, which may provide information related to the hazard associated with the use of both starting materials and cleaning agents, and the way the residues are effectively removed and detected.

Cleaning validation protocols should also take account of the personnel accessing the working rooms, including cleaning service personnel, and sampling and testing should be repeated for a reasonable number of times, considering the worst case in terms of number of persons entering the labs, of operations performed and of “hot spots” where cleaning may be more difficult for accessibility reasons (recesses, hidden parts of equipment / labs). Worst case approach might allow to “bracket” the different cleaning products and procedures, thus reducing the need for multiple validation protocols.

Cleaning validation protocol should be considered as a mean to validate cleaning procedures and cleaning media at the same time.

## Process validation

As already stated above, Process Validation (PV) should be viewed as the final step of validation, aimed to verify that the preparation process of a RP is capable to prepare the product with the requested characteristics of yield, quality, reliability, safety and efficacy, and that the RP is prepared within a suitable environment, with the necessary safety for the operating personnel and for the product. For the above reasons, it is expected that process validation is being performed when process design, and all the details of the process are adequately known. Preparation of test batches is usually of help and increase the probability of a successful PV. PV should be completed prior to the use of the intended RP in routine clinical activity, while this is not strictly required in case of investigational RPs, where it is considered the possible lack of well-established routine procedures. Objectives and acceptance criteria of PV should be clearly stated. In general, traditional approach to PV consists of the preparation of three consecutive batches of the intended RP, in different days and in the same conditions set for typical routine preparations. This means that starting activity of the radionuclide, reagents / solvents and their quantities, automated system parameters (if applicable), reaction, purification and formulation conditions should be the same as those intended for the routine preparation of the desired radiopharmaceutical. Moreover, the three productions should yield batches with the intended size (e.g. number of vials), and with appropriate labelling and packaging. Every batch of RP prepared accordingly with the above set of conditions should be fully characterized from the analytical point of view, with the aim to verify that the product meet the acceptance criteria as for all the established quality parameters (e.g. radiochemical purity, pH, sterility, radioactive concentration, etc.).

A PV protocol should include, but not necessarily limited, to:

- a description of the process to be validated, including the instrumentation used (e.g., automated radiosynthesis system, automated dispensing system, the hot cell where the automated devices are installed or where the preparation is manually performed, the dose calibrator, etc.), a preparation flow chart, including a list of reagents/solvents/excipients and related quantities, the proposed purification process (e.g. HPLC vs SPE, or both), if applicable, together with major components (e.g. detectors and separation conditions in case of HPLC purification, or the cartridges and method in case of SPE purification), the formulation and final sterilization methods of concern;
- a list of key personnel involved in validation activities, their functions and their training status;
- a list of the qualification protocols code numbers related to the various instruments which are used in the preparation process, together with the related qualification dates, with the aim to demonstrate that the above instruments status is compliant with the general validation policy;
- the list of the intended analytical tests and the related documentation, including analytical method validation protocols code numbers, if applicable, which are expected to be performed during the protocol execution;
- finished product release specifications;
- indication of the test to be performed after the release (e.g. sterility, radionuclidic purity);
- sampling plan, with indications of which, how many and when samples to be sent to Quality Control (QC) are collected, and selection criteria;
- a list of the deviations actually occurred (if any) during the execution of the tests, together with a discussion about their potential impact on the quality of the final product and the requested corrective action;
- a final discussion about the results (the Validation Summary Report).

Results obtained from PV help to monitor critical process parameters and their acceptance criteria / limits. In particular, radioactive concentration has to be considered as a better indicator /criteria than the amount of radioactivity as such. In case of RPs labelled with short or very short half-life radionuclides (e.g. C-11 or Ga-68), it might be difficult to comply with European Union (EU) guidelines, that often claim for radioactivity at Activity Reference Time (ART) to be defined for the final radiopharmaceutical product, and process validation is then used to establish a suitable radioactivity concentration range. Process validation is also aimed to define volume (or a range of volumes), which is another parameter that may be difficult, due to technical reasons, to univocally set in case RPs are prepared with an automated system, and no dispensing systems are available.

Retrospective validation, that is the process validation based on historical data of well-known and established preparation processes, is no longer considered an acceptable approach (Aerts et al. 2014).

An alternative approach, set by the European Medicine Agency (EMA) Guidelines on Process Validation (Guideline on process validation for finished products – information and data to be provided in regulatory submissions & EMA/CHMP/CVMP/QWP/749073/2016. http://www.ema.europa.eu/docs/en_GB/document_library/Scientific_guideline/2014/02/WC500162136.pdf. Accessed 31 Mar 2017), and by Annex 15 – GMP (EU) is the so called “Continuous Process Verification” (CPV), in which the continuous monitoring and evaluation of suitable parameters may replace the traditional process validation approach above described. CPV makes sense in case of well-known and fully developed preparation processes, and requires the monitoring of process performance and product quality on each batch of the intended (radio) pharmaceuticals. As these criteria are often met by the preparation of RPs, which are fully characterized before their release, this approach seems to be well suited and it may replace the need for re-validation, provided that the preparation process does not undergo significant changes.

## Validation of aseptic operations via Media fill

The objective of aseptic processing is to maintain the sterility of a product that is assembled from components, each of which has been sterilized by one of the methods described in Ph. Eur (European Pharmacopoeia). This is achieved by using conditions and facilities designed to prevent microbial contamination. In order to maintain the sterility of the components and the product during processing, careful attention needs to be given to: environment, personnel, critical surfaces, container / closure sterilization and transfer procedures, maximum holding period of the product before filling into the final container.

Aseptic operations may be validated by means of process simulation tests using microbial growth media, which are then incubated and examined for microbial contamination (**media fill tests**).

Most radiopharmaceuticals are designed for parenteral application and thus foresee operations to be performed under aseptic conditions. A media fill is the performance of an aseptic procedure mimicking the conditions of the real procedure, but using a sterile microbiological growth medium instead of the solutions otherwise used in the preparation of the radiopharmaceutical. The purpose of media fill procedure is to test whether the aseptic procedures are adequate to prevent contamination during actual RP production. Media fill may thus be considered as a part of the process validation of the RP preparation.

The media fill should evaluate the aseptic assembly and operation of the critical (sterile) equipment, qualify the operators and assess their technique, and demonstrate that the environmental controls are adequate to meet the basic requirements necessary to produce a sterile RP by aseptic processing (FDA Guidance).

The media fill should include positive control, which may be represented by a sealed product container of the growth medium inoculated with a small number of microorganisms, and a negative control, to ensure the absence of false positive results. A negative control may be prepared by pre-incubating the medium, or by aseptically transferring medium into a separate suitable sterile container and incubating the control simultaneously with the media fill test containers. The controls should be incubated under the same conditions as the media fill containers (EN ISO 14644 1 Cleanroom and associated controlled environments Part 1 classification of air cleanliness). Positive control test and growth promotion testing of the medium are usually performed by a commercial vendor or microbiology department of the hospital. In any case, inoculation of the positive control container is always performed in an area separated from the critical manufacturing area.

All steps in a media fill should be done in the same locations as those typical for the radiopharmaceutical production. To initially qualify an aseptic process at a specific facility, three media fills should be conducted on three separate days, following the procedures of the specific production process that is being qualified. Additionally, media fill should be conducted whenever significant changes are made to the aseptic process (e.g. changes in personnel, components, or equipment) and whenever there is evidence of a failure to maintain product sterility. Media fill tests performed to validate an aseptic process at a specific facility should be done by operators who have previously been trained and qualified in aseptic techniques (e.g., proper gowning, disinfection practices, handling sterile materials).

Media fills are an important element of operator qualification. To become a qualified operator for radiopharmaceutical product production, an operator should perform three media fills on three separate days. A qualified operator should perform a media fill at least annually (FDA Guidance).

During media fill test monitoring of the air, personnel and critical surfaces should be done.

## Additional file

Additional file 1: Additional information – practical examples. (DOCX 57 kb)

## Abbreviations

ART: Activity Reference Time
CFU: Colony Forming Unit
CLV: Cleaning Validation
CPP: Critical process parameters
CPV: Continuous Process Verification
CV: Coefficient of variation
DQ: Design Qualification
EANM: European Association of Nuclear Medicine
EMA: European Medicine Agency
EtOH: Ethanol
EU: European Union
FAT: Factory Acceptance Testing
FID: Flame ionisation detector
FDA: US Food and Drug Administration
GAMP: Good Automated Manufacturing Practice
GC: Gas chromatography
GMP: Good Manufacturing Practice
cGRPP: Current good radiopharmacy practice
HEPA: High Efficiency particulate air
HPGe: High purity germanium
HPLC: High-performance liquid chromatography
HVAC: Heating, Ventilation and Air Conditioning
ICH: International Conference on Harmonization of technical requirements for pharmaceuticals for human use
IMPD: Investigational Medicinal Product Dossier
IQ: Installation qualification
ISO: International Organisation for Standardization
LOD: Limit of detection
LOQ: Limit of quantitation
MDA: Minimum Detectable Activity
OQ: Operational qualifications
PET: Positron Emission Tomography
PIC/S Scheme: The Pharmaceutical Inspection Convention and Pharmaceutical Inspection Co-operation Scheme
Ph. Eur: European Pharmacopoeia
PLC: Programmable logic controller
PQ: Performance qualification
QC: Quality control
R^2^: Coefficient of determination
RP(s): Radiopharmaceutical(s)
R_s_: Resolution factor
RSD: Relative standard deviation
SAT: Site acceptance testing
SD: Standard deviation
SOP: Standard operating procedure
SPC: Summary of product characteristics
SPE: Solid phase extraction
SPECT: Single photon emission computed tomography
SSRP: Small-Scale “in-house” Radiopharmaceutical
TLC: Thin layer chromatography
TSB: Tryptic soy broth
UPS: Uninterruptible power supply
URS: User requirements specification
UV: Ultraviolet
VMP: Validation Master Plan
WHO: World Health Organization
