# Supplementary material for: Guidance on validation and qualification of processes and operations involving radiopharmaceuticals
Source: EJNMMI Radiopharm Chem. 2017 Jun 29;2:8. doi: 10.1186/s41181-017-0025-9 (PMC5824699; doi:10.1186/s41181-017-0025-9)
Supplement: Supplementary file 1 — Additional information – practical examples. (DOCX 57 kb) [file 41181_2017_25_MOESM1_ESM.docx]

**Additional Information – Practical Examples**

**Example 1: Qualification of a production instrument: automated system for the preparation of F-18 labelled radiopharmaceuticals**

Installation Qualification

***Aim***

- Verify installation status and utilities;
- Identify the critical components
- Check for dedicated SOPs aimed to use and maintenance of the system;
- Check for documentation (manuals, administrative documentation)
- Check for list of spare parts

***Description***

The system includes the following components:

- The radiosynthesis module;
- A UV detector;
- A semi-preparative HPLC pump;
- A control PC;
- A PLC.

***Test***

Test 1 : documentation

Please use the following form to collect data:

| **Document (e.g. manual, SOP) and title** | **Code** | **Location** |
| --- | --- | --- |
|  |  |  |
|  |  |  |
|  |  |  |
|  |  |  |
|  |  |  |
|  |  |  |
|  |  |  |
|  |  |  |
|  |  |  |
|  |  |  |

|  | **Yes** | **No** |
| --- | --- | --- |
| **CONFORM :** | ❒ | ❒ |

Test 2 – System description

| Radiosynthesis Module | | | | |
| --- | --- | --- | --- | --- |
| Components | | | | |
| **Description** | **Identification (ID#)** |  | **Installed** | |
| Radiosynthesis module |  |  | ❑ yes | ❑ No |
| PC |  |  | ❑ yes | ❑ No |
| PLC |  |  | ❑ yes | ❑ No |
| HPLC pump |  |  | ❑ yes | ❑ No |
| UV detector |  |  | ❑ yes | ❑ No |

For each component please collect the following information:

| **Radiosynthesis module** | |
| --- | --- |
| Identification |  |
| Supplier |  |
| Model |  |
| Serial number |  |
| Location (room) |  |
| Room temperature |  |
| Room relative humidity |  |

Operational Qualification

Test 1 – helium flow verification

***Aim***: to verify the efficiency of helium flow controller

***Methodology***:

- connect the calibrated flow meter in a proper position downstream with respect for the helium flow controller;
- using the control software, enter the desired helium flowrate value
- report the automated synthesis and calibrated flow meter output and record on the following data sheet.

***Acceptance criteria***: the difference between the two above output should not be > 2%

| **Recorded flow values** | | | | |
| --- | --- | --- | --- | --- |
|  | ***Test # 1*** | | ***Test # 2*** | |
| **Actual flow values (mL/Min)** | **Set values** | **Measured values** | \| **Set values** \| **Measured values** \| \| --- \| --- \| | \| **Set values** \| **Measured values** \| \| --- \| --- \| |
|  |  |  |  |  |
|  |  |  |  |  |
|  |  |  |  |  |
|  |  |  |  |  |
|  |  |  |  |  |
|  |  |  |  |  |

Test 2: reactor temperature verification

***Aim***: to verify that the temperature inside the reactor are consistent with the set values

***Methodology***:

- install the reactor following the normal procedure;
- place a calibrated thermocouple close to the following critical point: a) inside the reactor b) close to the automated system sensor;
- start a simulation of the intended RP preparation procedure, avoiding the use of radioactivity;
- wait until the automated procedure reach the critical reaction step (e.g. nucleophilic substitution reaction), which is usually carried out at 80°C for 10 minutes;
- record at defined time intervals (e.g. every 30 seconds) the actual temperature values;
- take a note of the measurement time;
- report experimental data in the following table.

***Acceptance criteria***: the average experimental temperatures should fall within the following range : 78-82°C.

| **Nucleophilic substitution Reaction temperatures** | | | | | | | |
| --- | --- | --- | --- | --- | --- | --- | --- |
|  | **Start reaction time (point a):**  **End reaction time (point a):** | | | | **Start reaction time (point b):**  **End reaction time (point b):** | | |
| # | Time | Thermocouple measured temperatures at point **a** | Automated system measured temperature | # | Time | Measured temperatures at point **b** | Automated system measured temperature |
|  |  |  |  |  |  |  |  |
|  |  |  |  |  |  |  |  |
|  |  |  |  |  |  |  |  |
|  |  |  |  |  |  |  |  |
|  |  |  |  |  |  |  |  |
|  |  |  |  |  |  |  |  |
|  |  |  |  |  |  |  |  |

Performance Qualification

***Process verification test***

***Aim***: to verify that the preparation process of the intended radiopharmaceutical is performed correctly.

***Methodology***:

- prepare the automated synthesis system following the normal SOP dedicated to the preparation of the intended radiopharmaceutical, loading reagents / solvents / components in the proper quantities and following the desired order;
- perform a full preparation process until the final product is collected in the proper container;
- record and report the data obtained during the preparation in the following data sheet;
- repeat the preparation process three times.

***Acceptance criteria***:

The preparation of the desired radiopharmaceutical has to be performed correctly, meeting specification and acceptance criteria for the various parameters, without significant deviations. The process may be split in the following steps:

- ^18^F-fluoride transfer from the target to the automated synthesis system; transferred radioactivity is measured;
- purification / trapping of ^18^F-fluoride using QMA cartridge;
- nucleophilic substitution reaction;
- hydrolysis;
- SPE purification;
- sterilization using membrane filters;
- final formulation;
- activity of final product should fall within the following interval : 2-8GBq;
- radioactive concentration should meet the following criteria : 100 – 500MBq/mL;
- the volume of the final product solution should be 15-20 mL.

| **performance qualification test** | | | | | | | | | | | |
| --- | --- | --- | --- | --- | --- | --- | --- | --- | --- | --- | --- |
| **Automated synthesis system model …..** | | | | | | | | | | | |
| **Batch number** | | |  | | | | | **Date:** | | |  |
| *^18^F-fluoride* ***transfer*** | | | | | | | | | | | |
| Measured transfer time: | | ___________ min | | | Expected transfer time: | | | | | max. 5 min | |
| The F-18 transfer step was performed as expected | | | | | | | | | | ❒Yes❒ NO | |
| Detected F-18 activity: | | | | | | | | | | _______ MBq | |
| ***Purification / trapping of*** *^18^F-fluoride* ***using QMA cartridge*** | | | | | | | | | | | |
| Detected activity after purification using QMA: | | | | | | | | | | **_______**MBq | |
| Specification: | ≥ 80% of starting the activity has been recovered | | | | | | | | Conform: | ❒Yes❒ NO | |
| ***Nucleophilic substitution reaction*** | | | | | | | | | | | |
| Desired reactor temperature | | | | 100 °C | | Actual temperature: | | | | ______ °C | |
| Detected activity at the end of the nucleophilic substitution reaction : | | | | | | | | | | _______ MBq | |
| ***Hydrolysis*** | | | | | | | | | | | |
| Desired reactor temperature: | | | | 110°C | | | Actual temperature | | | ______°C | |
| Detected activity at the end of hydrolysis step: | | | | | | | | | | ____________ MBq | |

| ***Semi-preparative HPLC purification*** | |
| --- | --- |
| Retention time of the desired product is: |  |
| Specification: 15-17 min |  |
| ***Sterilization and final formulation*** | |
| The solution correctly passes through the filters | ❒Yes❒ NO |
| The vial is correctly transferred to the dose calibrator | ❒Yes❒ NO |
| Detected final product activity | ______MBq |
| Specification: 2-8 GBq | ❒Yes❒ NO |

**Example 2: Process Validation**

***Aim***: to ensure that the preparation process of the radiopharmaceutical [^18^F]123 yield the intended product with characteristics suitable for its use in clinical diagnostic routine.

Process validation requires the preparation and quality control of three batches of the radiopharmaceutical [^18^F]123, following the same procedures, using the same instruments, and using the same reagents/solvents/ components in the same amounts and characteristics intended for the routine preparation of t[^18^F]123.

***Description of the preparation process***: [^18^F]123 is prepared via the following steps:

1. All the required starting materials need to be ready;
2. Aseptic assembly, in a laminar flow cabinet, of the final container (sterile, pyrogen free, 15 ml vial) with sterilizing filter, vent filter and sterile needles;
3. radiosynthesis, purification and sterilization of the desired radiopharmaceutical, from the production of the radionuclide F-18, until the final sterile, pyrogen free solution of [^18^F]123 is formulated in the final container;
4. labelling of the container (vial) and of the secondary container (shielded lead container);
5. sampling and QC tests;

Here the process has been described in brief; in the real validation protocol it should be more detailed.

***Tests***

*Preparation of [^18^F]123*

Prepare three batches of [^18^F]123, following the indications described in the proper SOPs. Report the experimental data in the following data sheet.

| ***Radiopharmaceutical: [^18^F]123*** | | | |
| --- | --- | --- | --- |
| **Name and address of the preparation facility** | | **Module # 002** | |
| **Pharmaceutical form:** | | **Injectable solution** | |
| **Date of preparation:** | **Preparation time:** | | **Batch n°:** |

| **Step 1: cyclotron production of F-18** | |
| --- | --- |
| 1.1. starting beam time: | 1.2. end of irradiation time (EOB): |
| 1.3. Target n°: | 1.4. target average current: |
| 1.5. Target integrated current : | 1.6. Detected F-18 radioactivity* |
| 1.7 Annexes : irradiation data sheet | |
| 1.8. Comments: *F-18activity is determined by a built-in probe of the automated system | |
| 1.9. Operator: | 1.10. Operator’s signature : |

| **Step2: preparing the automated radiosynthesis system** | | | |
| --- | --- | --- | --- |
| 2.1 Reactor leak test (leak rate ≤ 5 kPa/min) Conform ❑Yes❑ NO | | | |
| 2.3 List of starting materials: | Amount | Batch N. | Expiry date |
| [^18^O]H_2_O | 2 g |  |  |
| Kryptofix | 15 mg / 1 ml acetonitrile |  |  |
| K_2_CO_3_ | 3.5 mg / 0.5 ml water for injection (wfi)acquap.p.i. |  |  |
| Precursor | 10 mg |  |  |
| Acetonitrile | 1 ml |  |  |
| HCl 1M | 0.3 ml |  |  |
| WFI | 5 ml |  |  |
| HPLC mobile phase (wfi / acetonitrile; 95/5) | 1 L |  |  |
| QMA-light cartridge | 1 |  |  |
| 2.4 Shielded lead container is ready : ❑Yes❑ NO | | | |
| 2.5 Label for shielded container is ready: ❑Yes❑ NO | | | |
| 2.6 Comments | | | |
| 2.7 Operator: | 2.8 Operator’s signature: | | |

| **Step 3: assembly of sterile vial + filters** | | | |
| --- | --- | --- | --- |
| 3.1. Laminar flow(m/s): | Limit (m/s): 0.36÷0.54 | Conform: Yes NO | |
| 3.2 Materials: | Amount | Batch N. | Expiry |
| Sterile vials | 1 |  |  |
| Sterilizing filter | 1 |  |  |
| Venting filter | 1 |  |  |
| Needles 22G x 1/4" (0.70 x 30 mm) | 2 |  |  |
| 3.3 label for vial is ready: Yes NO | | | |
| 3.4 Operator: | 3.5 Operator’s signature: | | |

| **Step 4: radiosynthesis of [^18^F]123** | | |
| --- | --- | --- |
| 4.1. Activity at start of synthesis(after elution of QMA), in MBq: | | |
| 4.2. Start synthesis time: | |  |
| 4.3. Activity at the end of synthesis (EOS) in MBq: | | |
| 4.4. EOS time: | | |
| 4.5. Radiochemical yield% (not corrected for decay): | | |
| 4.6. Expiry time: | | |
| 4.7. Annexes: automated synthesis module report printout | | |
| 4.8. Operator | 4.9. Operator’s signature | |

| **Acceptance criteria** | |
| --- | --- |
| Radioactive concentration: 200-400MBq/Ml Conform: Yes NO | |
| Final volume : 8-12 mL Conform: Yes NO | |
| Resp. of the preparation: | Resp. of the preparation’s signature : |

***Quality control of the prepared batches of [^18^F]123***

Batches of [^18^F]123 are controlled following approved and validated (if applicable) analytical methods implemented during radiopharmaceutical development.

| **CERTIFICATE OF ANALYSIS** | | | | | | **MOD. # 003** | | |
| --- | --- | --- | --- | --- | --- | --- | --- | --- |
| **Radiopharmaceutical** | | | **[^18^F]123** | | | | | |
| **Date** |  | | **Batch number** | |  | | | |
| **Analysis** | **results** | **Acceptance criteria** | | **date** | | | **Conform** | |
| pH |  | 4.5 – 8.5 | |  | | | **Yes** | **NO** |
| Appearance |  | Clear, colourless solution | |  | | | **Yes** | **NO** |
| Identification: gamma spectrometry |  | 511 ± 10 KeV | |  | | | **Yes** | **NO** |
| Identification: HPLC |  | Main radioactive peak has retention time similar to that of the cold standard of [^19^F]123 | |  | | | **Yes** | **NO** |
| Residual solvents: acetonitrile |  | ≤ 4.1 mg/V* | |  | | | **Yes** | **NO** |
| Residual solvents: acetone |  | ≤ 50 mg/V* | |  | | | **Yes** | **NO** |
| Chemical purity: 123 |  | ≤ 20μg/V* | |  | | | **Yes** | **NO** |
| Chemical purity: K222 |  | ≤ 2.2 mg/V* | |  | | | **Yes** | **NO** |
| Other impurities |  | 0.5 mg/V* is the sum of « other » impurities | |  | | | **Yes** | **NO** |
| Radiochemical purity: HPLC |  | ≥ 95% di [^18^F]123 | |  | | | **Yes** | **NO** |
| Radionuclidic purity: gamma |  | F-18 > 99.9% | |  | | | **Yes** | **NO** |
| Half-life |  | 105-115 min | |  | | | **Yes** | **NO** |
| Sterility |  | Sterile (Ph. Eur.) | |  | | | **Yes** | **NO** |
| Bacterial endotoxins |  | < 175 IU/V (Ph. Eur.) | |  | | | **Yes** | **NO** |

* V = maximum recommended dose in millilitres

***Conclusions***

Validation protocol of the radiopharmaceutical [^18^F]123 has been carried out satisfactorily. Radiochemical yield, radioactive concentration and quality of the desired product always met all the acceptance criteria, and no deviations or out of specifications were observed during the execution of the tests. Thus, the preparation process [^18^F]123 showed to be reliable and effective. In conclusion, preparation process of [^18^F]123 is suitable for its intended use in routine diagnostic activity using PET/CT.

**Example 3:** **Validation of operating personnel with Media fill test**

1. ***Purpose***

This operating procedure applies for validation and periodic revalidation of the process of aseptic preparation and dispensing of radiopharmaceuticals obtained from radionuclide precursor and kit for radiopharmaceutical preparation. It is also intended for qualification and requalification of the personnel working in the aseptic preparation of radiopharmaceuticals.

1. ***General requirements***

All steps in a media fill should be done in the same locations as the radiopharmaceutical production steps. To initially qualify an aseptic process at a specific facility, **three media fills should be conducted on three separate days**. The manipulation of materials such as vials and disposables should be done in a same manner as usually used in the hot-lab (i.e. sanitization of the material before the introduction in the work area; sanitization of the rubber stopper of the bottles prior to sampling, using syringe and vials shielding, taking into account measuring steps, incubation step, etc.).

Although during the media fill simulation there is no radioactivity, it is essential to act accordingly with the rules adopted in daily routine radioprotection, since you must reproduce the methods used in the daily routine.

During media fill test monitoring of the air, personnel and critical surfaces should be done (according to SOP: Microbiological monitoring).

To validate or revalidate the process execution of three simulations is necessary.

The culture media should support growth of a wide range of microorganisms (fungi, yeasts and aerobic bacteria) such as the TSB (Tryptic soy broth) medium distributed in bottles needed for the media fill test.

After the test, at least 2 mL of the media should be left in each vial, which enables sterility testing.

Check at the end of the simulation the absence of radioactive contamination of the vials, before shipment to the laboratory for incubation.

1. ***Materials:***

| TSB solution 20 mL in 25 mL vial | 1 vial |
| --- | --- |
| TSB solution 10 mL in 12 mL vial | 1 vial |
| Sterile Vacuum vials 10 mL | 1 + 4 = 5 vials |
| Syringe 10 mL | 1 syringe |
| Syringe 2.5 mL | 4 syringes |
| Syringe 1 mL | 1 syringe |
| Needles 23 G | qs |
| Agar plates | as prescribed in SOP (Microbiological monitoring) |
| Rodac plates | as prescribed in SOP (Microbiological monitoring) |
| Swabs for microbiological monitoring | as prescribed in SOP (Microbiological monitoring) |

TSB (Tryptic Soy Broth) is obtained from Microbiology department, where the growth promotion test according to Eur. Ph. is performed and positive and negative control solutions prepared.

1. ***Operating Procedure***

|  |  |
| --- | --- |
| TSB solution 20 mL in 25 mL vial | **S1** |
| TSB solution 10 mL in 12 mL vial | **R1** |
| Sterile Vacuum vials 10 mL | **A1;**  **P1-1, P1-2, P1-3, P1-4** |
| Syringe 10 mL | 1 syringe |
| Syringe 2.5 mL | 4 syringes |
| Syringe 1 mL | 1 syringe |

1. Prepare all material needed for performing the media fill test, and carefully sanitize it and put it in to the material air-lock as required by specific SOP (Introduction of the material into the clean rooms);
2. enter into the hot-lab (laboratory for the preparation of radiopharmaceuticals). Wear appropriate clothing, cap, mask and gloves as required by specific SOP (Entry of personnel in clean rooms);
3. transfer all needed materials into the laminar-flow cell, use appropriate shielding as when working with radioactivity;
4. make a note of the batch record all lot numbers of expiration dates;
5. label the vials according to the scheme above:

**S1**: Bottle containing 20 mL of TSB media - Simulates the saline used to dilute the radionuclide to the required concentration for the reconstitution of the kit for radiopharmaceutical preparation (lyophilized) - used in the simulation 1

**S2**: Bottle containing 20 mL of TSB media - Simulates the saline used to dilute the radionuclide to the required concentration for the reconstitution of the kit for radiopharmaceutical preparation (lyophilized) - used in the simulation 2, ***second day***

**S3**: Bottle containing 20 mL of TSB media - Simulates the saline used to dilute the radionuclide to the required concentration for the reconstitution of the kit for radiopharmaceutical preparation (lyophilized) - used in the simulation 3, ***third day***

**R1**: Bottle containing 10 mL of TSB media - Simulates the solution of radiopharmaceutical precursor used to radio-label the kit for radiopharmaceutical preparation - used in the simulation 1

**R2**: Bottle containing 10 mL of TSB media - Simulates the solution of radiopharmaceutical precursor used to radio-label the kit for radiopharmaceutical preparation - used in the simulation 2, second day

**R3**: Bottle containing 10 mL of TSB media - Simulates the solution of radiopharmaceutical precursor used to radio-label the kit for radiopharmaceutical preparation - used in the simulation 3, third day

**A1**: 10mL bottle - Simulates the kit for radiopharmaceutical preparation (containing the lyophilized drug to be radiolabeled) in the simulation 1

**A2**: 10mL bottle - Simulates the kit for radiopharmaceutical preparation (containing the lyophilized drug to be radiolabeled) in the simulation 2, ***second day***

**A3**: 10mL bottle - Simulates the kit for radiopharmaceutical preparation (containing the lyophilized drug to be radiolabeled) in the simulation 3, ***third day***

**P1-1**: 10mL bottle - Simulates the dispensing step for patient 1 in the simulation 1

**P1-2**: 10mL bottle - Simulates the dispensing step for patient 2 in the simulation 1

**P1-3**: 10mL bottle - Simulates the dispensing step for patient 3 in the simulation 1

**P1-4**: 10mL bottle - Simulates the dispensing step for patient 4 in the simulation 1

***Second day***

**P2-1**: 10mL bottle - Simulates the dispensing step for patient 1 in the simulation 2

**P2-2**: 10mL bottle - Simulates the dispensing step for patient 2 in the simulation 2

**P2-3**: 10mL bottle - Simulates the dispensing step for patient 3 in the simulation 2

**P2-4**: 10mL bottle - Simulates the dispensing step for patient 4 in the simulation 2

***Third day***

**P3-1**: 10mL bottle - Simulates the dispensing step for patient 1 in the simulation 3

**P3-2**: 10mL bottle - Simulates the dispensing step for patient 2 in the simulation 3

**P3-3**: 10mL bottle - Simulates the dispensing step for patient 3 in the simulation 3

**P3-4**: 10mL bottle - Simulates the dispensing step for patient 4 in the simulation 3

1. Introduce within the LAF all the material needed to perform the first simulation: **S1, R1, A1, P1-1, P1-2, P1-3, P1-4**
2. Insert the bottle R1 in the radionuclide calibrator to simulate the activity measurement of the eluate. Put it back into the led vial shield
3. Take a 10 mL syringe, use syringe shield withdraw 2 mL of the solution from the **R1** vial and dilute to 10 mL with the solution from the **S1** vial
4. Insert the syringe containing 2 mL of TSB from activity vial **R1** and 8 mL of TSB from the saline vial **S1** in the radionuclide calibrator to simulate activity measurement in the syringe.
5. Use syringe shield to transfer the volume from the syringe inside the kit vial - **A1 (shielded).**
6. Shake the vial, insert the kit vial - **A1** in the radionuclide calibrator to simulate activity measurement of the final product.
7. Simulate the incubation step for 10 minutes on ambient temperature (20-25 °C).
8. Simulating the cooling phase for 5 minutes.
9. Take 1 mL syringe and withdraw 0,3 mL of solution – this is to simulate the aseptic sampling intended to perform QC.
10. Dispense the content of the vial A1 to 4 patient doses as follows:

**P1-1**:

- - With a 2.5 sterile syringe Withdraw 2 mL of the solution from the vial **A1**
  - Measure the activity in the radionuclide calibrator
  - Transfer the contents of the syringe into the vial **P1-1**

**P1-2**:

- - With a 2.5 sterile syringe Withdraw 2 mL of the solution from the vial **A1**
  - Measure the activity in the radionuclide calibrator
  - Transfer the contents of the syringe into the vial **P1-2**

**P1-3**:

- - With a 2.5 sterile syringe Withdraw 2 mL of the solution from the vial **A1**
  - Measure the activity in the radionuclide calibrator
  - Transfer the contents of the syringe into the vial **P1-3**

**P1-4**:

- - With a 2.5 sterile syringe Withdraw 2 mL of the solution from the vial **A1**
  - Measure the activity in the radionuclide calibrator
  - Transfer the contents of the syringe into the vial **P1-4**

1. Remove all the material used during the first simulation. Fill in the form for sterility testing and send vials S1, R1, A1, P1-1, P1-2, P1-3 and P1-4 to the Microbiology department
2. For second and third simulation repeat steps from 5-17.

***Conformity***

Sterility testing is performed at Microbiology department. Save results from the sterility testing together with the batch record. The media-fill test conforms to the requirements if all samples in all three runs are marked as Sterile.
